# Measuring the nature and duration of symptoms of cervical cancer in young women: developing an interview-based approach

**DOI:** 10.1186/1472-6874-13-45

**Published:** 2013-11-13

**Authors:** Anita WW Lim, Lindsay JL Forbes, Adam N Rosenthal, Kantipati S Raju, Amanda-Jane Ramirez

**Affiliations:** 1 Kings College London Promoting Early Presentation Group, 9th Floor Capital House, 42 Weston Street, London SE1 3QD, UK; 2 Centre for Cancer Prevention, Wolfson Institute of Preventive Medicine, Barts & The London School of Medicine and Dentistry, Queen Mary University of London, Charterhouse Square, London EC1M 6BQ, UK; 3 Barts Cancer Institute CR UK Centre of Excellence, London, UK; 4 Gynaecological Oncology, St Thomas Hospital, Guy’s and St Thomas’ NHS Foundation Trust, London, UK

**Keywords:** Cervical cancer, Early diagnosis, Symptoms

## Abstract

**Background:**

Some young women experience delays in diagnosis of cervical cancer, but little research about ways of studying these delays has been published. A major challenge is that gynaecological symptoms are common in young women, but cervical cancer is rare. This study describes the development and testing of a measure for studying delays in diagnosis in young women with cervical cancer.

**Methods:**

Prospective development of an interview measure and testing of its ability to reliably and systematically collect relevant data in two large hospitals in London, UK using 27 women aged 18–40 diagnosed with cervical cancer in the previous two years. We developed a semi-structured interview schedule and data extraction form to systematically collect data on symptoms (including nature and duration) and risk factors for delayed diagnosis from young women with cervical cancer. We piloted the measure among young women with cervical cancer (audiorecording it with their permission), refining it iteratively. To complete the measure, we developed a database for managing the data and a manual for using the schedule. Two researchers extracted data from the recorded interviews to assess inter-rater reliability.

**Results:**

The final interview schedule yielded quantitative data on the nature and duration of symptoms and risk factors for delayed diagnosis. Inter-rater reliability was high. In the pilot, 12 of the 27 women were diagnosed via symptomatic presentation. Median time from the symptom triggering presentation to presentation was one month (interquartile range 0–4 months). Median time from presentation to diagnosis was three months (interquartile range 1–8.5 months).

**Conclusions:**

We have developed a reliable tool for measuring the nature and duration of symptoms in young women with cervical cancer. Pilot data suggest that a substantial proportion of women experience delay between first presentation and diagnosis.

## Background

Women under the age of 25 are not offered routine cervical screening in England because of the lack of evidence that screening reduces rates of invasive cancer in this age group, and the potential harms of unnecessary treatment [1,2]. While cervical cancer is rare in this age group (up to about 60 cases a year in England) [3] there have been reports that a significant proportion of these young women experience symptoms for some time prior to diagnosis and significant delays in diagnosis [4], which are thought to be because either the women or their health professionals do not recognise the seriousness of symptoms. Encouraging early presentation and prompt referral may reduce avoidable deaths and allow for fertility-sparing treatment options.

Young women commonly present in primary care with vaginal bleeding, including intermenstrual or postcoital bleeding, and vaginal discharge [5] and these are far more likely to be due to contraceptive side-effects or other conditions such as infections or cervical ectopy than cancer [5-7]. This means that approaches to encourage early presentation and prompt referral of women with symptoms suggestive of cervical cancer must be carefully designed to avoid unnecessary anxiety, primary care attendances, referrals and investigations. To inform these approaches we need a better understanding of the symptoms experienced by young women with cervical cancer, what influences the time they take to seek help and how the symptoms are managed in primary care. To our knowledge, no universally accepted, reliable research instruments for collecting systematic data about the history of presentation of women with cervical cancer have been published.

Collecting data on the nature and duration of symptoms of cancer is challenging because it is necessarily collected from people recently diagnosed with cancer, who may be feeling unwell or distressed and may be attending for treatment. Extracting data from medical records is unreliable because health professionals may under-record symptoms and may not record onset dates [8]. Researchers have used semi-structured and in-depth interviews (telephone or face-to-face) [9-11], and self-complete questionnaires to collect these data [11,12]. Semi-structured interviews have been shown, when collecting data on nature and duration of symptoms in other cancer types, to be feasible and acceptable, and to provide detailed and robust data [9,10]. Although interviews are more time-consuming and expensive, they generally produce higher quality data than self-complete questionnaires because they allow the interviewer to check data and probe for information that people might not have considered relevant. They may also be less distressing for people dealing with the implications of a cancer diagnosis and going through anticancer treatment. We have interviewed over 300 women with breast cancer using this approach, with a very low refusal rate and no serious adverse events [9,10].

The special challenges of collecting data from young women with cervical cancer are that they are likely to be particularly vulnerable because of their age and because some may be facing difficult treatment decisions involving a threat to fertility. Many will be living in socioeconomically deprived circumstances [3,13].

The aim of this study was to develop and test a reliable interview-based measure of the nature and duration of cervical cancer symptoms and the risk factors for delayed diagnosis, to be used in a prospective study of all young women diagnosed with cervical cancer over a one-year period, and which in turn would inform approaches to promoting early presentation and prompt onward referral.

## Methods

### Development of a measure of the nature and duration of cervical cancer symptoms

The measure comprised a semi-structured interview schedule, a data extraction form, a database and a manual. This was similar to a measure that we developed to examine the nature and duration of symptoms in breast cancer [9,10], and drew on published studies and national guidelines about cervical symptoms [14-22]. We also conducted a focus group to inform the schedule with four women who had been diagnosed with cervical cancer in their early thirties.

The purpose of the interview manual was to provide guidance on how to deliver the interview schedule and extract data. The data extraction form was used to record data during the interview. These data were entered onto the database for storage and analysis.

We structured the interview to promote accurate recall. The interviewer established a rapport with the woman and allowed the woman to describe key events at her own pace and in her own way. The woman was first asked to recount the story of the events leading to their diagnosis using open questions. The interviewer then probed to clarify the nature of symptoms including what the woman thought they were due to, and where the woman had presented to, if relevant. At this stage, the interviewer read out a list of symptoms to the woman, asking if she had experienced any of those in addition to symptoms previously reported. Symptom checklists have been shown to elicit symptoms occurring earlier in the history of presentation among cancer patients [23].

The interviewer used closed questions to collect data on dates of onset of symptoms and dates of healthcare attendances. To promote accurate recall of dates, the interviewer drew up a calendar timeline working with the woman to establish the correct chronology [24]. If the woman could not remember the date or timepoint of a symptom or healthcare attendance, the interviewer asked the woman to recall memorable events around that time such as birthdays, holidays or starting a new job, and asked the woman when the symptom or healthcare attendance occurred in relation to this, a technique known as calendar anchoring [25].

If the woman could not provide a date for a particular symptom onset or other event, the interviewer asked her to identify the month and year or a range of months for when it occurred, and whether it was early, middle or late in the month. If the woman was unable to recall even a range of months, the interviewer asked her to identify the season and whether it was early, middle or end of the season. If the woman could not recall any approximate dates, the interviewer asked if she could recall how long she had had the symptom or experienced the event before diagnosis.

One of the challenges in designing the measure was to categorise women’s pathways to diagnosis: women with cervical cancer may have presented many times before receiving the diagnosis, and there may have been referrals between secondary care specialties. A particular challenge was to define which symptoms were relevant in a reproducible way; women are likely to have experienced several gynaecological symptoms (some possibly caused by cancer, and some not) and we considered it important to make sure that we recorded the most relevant symptoms. Another challenge was to identify reproducibly the key healthcare attendances forming part of the pathway to diagnosis, because the women may have attended many times. We therefore developed definitions for pathways to diagnosis, key symptom types and key healthcare attendances.

### Pathways to diagnosis

**Figure 1:**
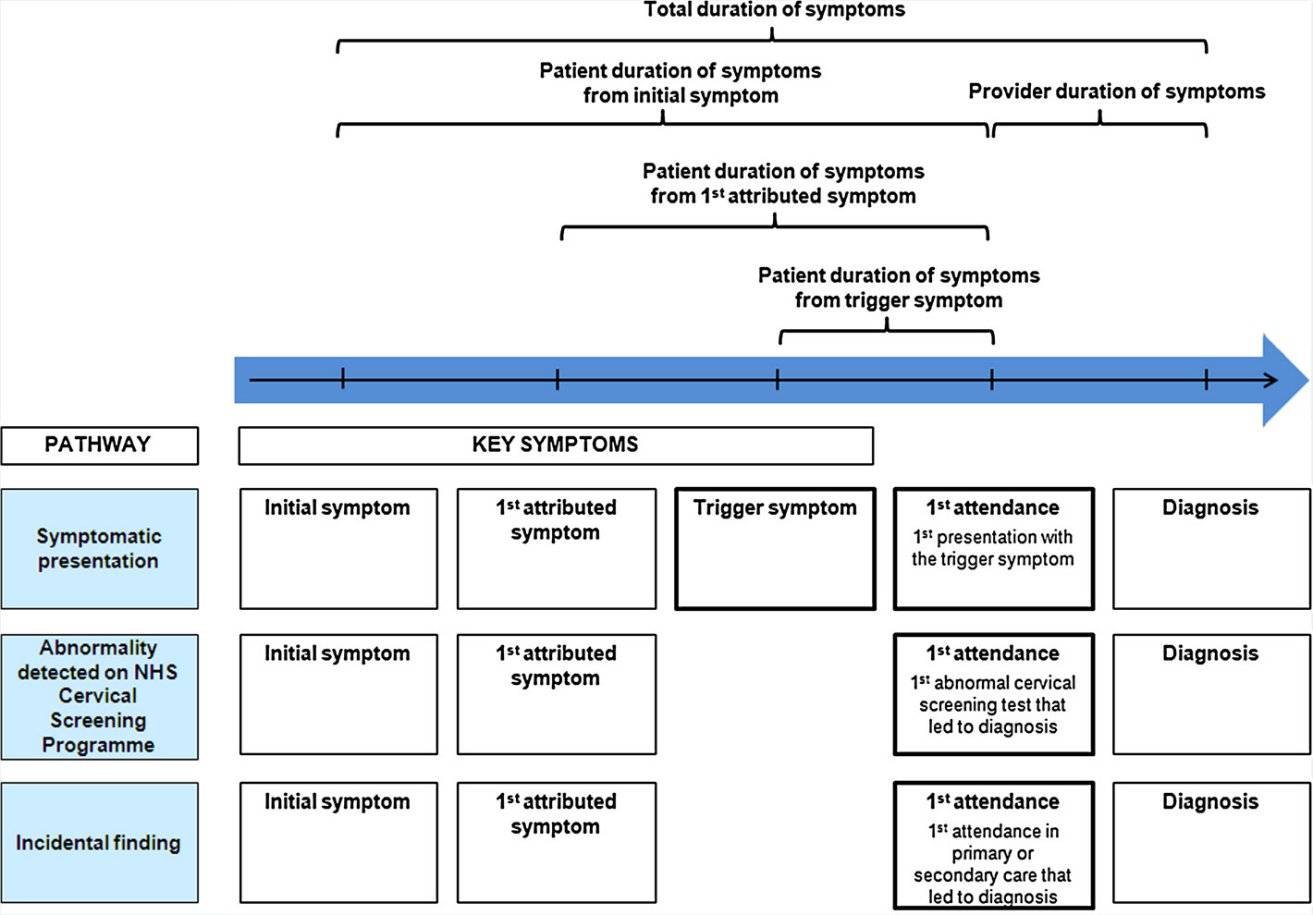
Pathways to diagnosis and key definitions.

Each woman was categorised according to her pathway to diagnosis (see Figure 1):

• Symptomatic presentation – women who presented to a health professional (doctor, nurse or midwife) with one of a list of symptoms that could be due to cervical cancer (known as 'checklist symptoms’) (see Additional file 1: Box 1) and that presentation eventually led to diagnosis (this included women diagnosed via abnormal cytology if the health professional carried out a cervical screening test as part of management of the symptom). We drew up the checklist drawing on published literature [17,19-22,26] and the Department of Health key messages about cervical cancer symptoms [27].

• Abnormality detected on National Health Service (NHS) Cervical Screening Programme – women who were diagnosed as a result of an abnormal finding (abnormal cytology or cervical pathology) on cervical screening following a routine invitation on the NHS Cervical Screening Programme (including women who attended screening because of their reported symptoms).

• Incidental finding – women who were diagnosed during procedures or investigations for another condition or problem (e.g. a suspicious lesion seen during insertion of an intrauterine device).

### Symptom types

We defined three key symptom types:

• the 'trigger’ symptom - the earliest checklist symptom that led the woman to present to a health professional and that led to diagnosis, irrespective of whether the woman thought it was caused by cervical cancer or not. The trigger symptom was usually the symptom elicited in response to the question; “What was the symptom that first prompted you to see a doctor or nurse?”

• the 'first attributed’ symptom - the symptom that the woman believed to be the first symptom of cancer at the time of the interview. The first attributed symptom was usually the symptom the woman reported in answer to the question; “Looking back now, what would you say was the first sign or symptom of your cancer?” This approach to defining the first symptom of cancer when collecting data retrospectively has been used in some other studies examining time to diagnosis in cancer [28]. For example, a woman may describe attending her GP because she had intermenstrual bleeding, but at the time of interview, looking back, she may believe that postcoital bleeding that she had two months earlier was due to her cervical cancer. For women diagnosed via symptomatic presentation, the trigger symptom may also have been the first attributed symptom.

• the 'initial’ symptom - the first checklist symptom that a woman reported, but did not attribute to cervical cancer at the time of the interview. For example, the same woman above may report vaginal discharge that started before her postcoital bleeding, that she attributes to a genital infection.

### Healthcare attendances

We defined two key health care attendances: the first attendance and date of diagnosis. Date of diagnosis was defined as the attendance on which the woman was told she had cervical cancer (for all women). The first attendance was defined as:

• for women diagnosed via symptomatic presentation, the first symptomatic presentation for a 'trigger’ symptom;

• for women diagnosed via the NHS Cervical Screening Programme, the attendance on which the first abnormal cervical screening test that led to diagnosis was carried out;

• for women diagnosed following an incidental finding, the first attendance in primary or secondary care that led to diagnosis.

### Risk factors for delay

We collected data on risk factors for delay in presentation, including nature of symptoms, whether or not the woman thought the symptom was serious at the time, whether she had children, age of leaving full-time education, contraceptive use, reported barriers to symptomatic presentation, and prior knowledge of cervical cancer. Most of these were added in the latter stages of interview measure development and were not analysed.

### Testing the measure

To test and further develop the measure, we performed audiorecorded interviews with women aged 18–40 diagnosed with cervical cancer in the previous two years, recruited through two London hospitals. Women up to age 40 were included for pragmatic reasons - cervical cancer is rare in women aged <25. Potentially eligible women were identified from a database of women diagnosed with gynaecological cancers at each hospital and were invited to participate via letter. The measure was refined iteratively after each interview, based on review, discussion and feedback from a professor of liaison psychiatry experienced in conducting research interviews with cancer patients (AJR).

Ethics approval was granted by the South West London REC3 Ethics Committee. All women provided written informed consent. Interviews were performed by the same interviewer (AWWL) either in the woman’s home or in a private room at the hospital.

To assess interview acceptability, we asked the women whether they found the questions difficult to understand or upsetting, and how difficult they found it to remember the details of the events.

We assessed inter-rater reliability by calculating percentage agreement for key interview data for four interviews. Two raters listened to interview transcripts and recorded the nature, severity and attribution of key symptoms, and the dates of key symptom onset and healthcare attendances.

### The Cervical Cancer History of Presentation Schedule (CCHOPS)

The interview schedule (see Additional file 2: Appendix 1) included three main domains:

• Symptoms – nature, duration, frequency, severity, attribution, actions prompted and disclosure to others

• Timepoints – key symptoms, key healthcare attendances

• Risk factors – sociodemographic factors, other health problems, contraception, perception of primary care, barriers to seeking GP help, cervical screening history, prior knowledge of cervical cancer (see Additional file 3: Box 2)

The final interview-based measure (Cervical Cancer History of Presentation Schedule (CCHOPS)) comprised:

• a semi-structured interview schedule (see Additional file 2: Appendix 1) – providing a flexible script for the interviewer that allows for probing and clarification of complex histories of presentation,

• a data extraction form - for recording the data items during the interview (available from the researchers),

• a database – for entering and storing data from the data extraction form (available from the researchers),

• a manual for interviewers - providing detailed guidance on how to deliver the schedule and extract data (available from the researchers).

### Analysis

A set of rules was used to assign calendar dates for all approximate timepoints (available from the researchers). Patient and provider durations of symptoms were calculated using figures rounded to the nearest month to avoid implying accuracy that may be spurious. We calculated medians and interquartile ranges of two types of duration of symptoms:

• patient-related duration of symptoms, which was the time from symptom onset (for all three symptom types) to first attendance,

• provider-related duration of symptoms, which was the time from first attendance to diagnosis (this therefore included the time before onward referral by primary care, and the time spent reaching the diagnosis in secondary care).

We defined patient delay as a duration of symptoms of three or more months from each key symptom to first attendance, and provider delay as duration from first attendance to diagnosis of three or more months, which is the convention in the cancer literature [29-31].

All statistical analyses were performed using STATA for Windows (version 11.0, StataCorp LP, College Station, TX).

## Results

### Testing the Cervical Cancer History of Presentation Schedule (CCHOPS) interview-measure and the resultant exemplar data

Table 1 shows demographic and clinical details of study participants. We performed interviews with 27 women, eight in the woman’s home and 19 in a private room at a hospital. Interviews lasted an average of 35 minutes (range 11 to 72 minutes).

**Table 1:** Demographic and clinical characteristics of participants

	**Number of participants (n = 27)**
**Age at diagnosis**	
<25	2
25-29	8
30-34	7
≥35	10
**Ethnicity**	
White British or White other	22
Other	5
**Living arrangements**	
Lives with partner/husband ± children	15
Lives with parents	2
Lives with children only	1
Lives with flatmate or alone	7
Missing or refused	2
**Age left full-time education**	
≤16	8
17-18	3
19+ or still studying	16
**Cancer stage at diagnosis (FIGO)**	
1a	13
1b	9
2	4
3	0
4	1
**Cancer histology**	
Squamous cell carcinoma	20
Adenocarcinoma	7

The women’s median age at diagnosis was 34 years (interquartile range 28 to 38 years). Two women were aged under 25 at diagnosis (one 23 and one 24). Most were White and just over half lived with a partner or husband. Nearly a third had left school at age 16 or earlier. Twenty-two women had International Federation of Gynecology and Obstetrics (FIGO) stage 1 cervical cancer at diagnosis (nine stage 1a (microinvasive disease)), four stage 2 and one stage 4.

### Pathways to diagnosis

Twelve women were diagnosed via symptomatic presentation: 11 presented to their GP surgery and one to a sexual health clinic. Twelve women were diagnosed following abnormal cytology or suspicious lesions found during routine screening on the NHS Cervical Screening Programme. Three women were diagnosed following an incidental finding: one during an emergency hysterectomy for post-partum haemorrhage, one during emergency Caesarean section and one after presenting to a sexual health clinic for a sexually transmitted infection screen.

### Women diagnosed via symptomatic presentation

Of the 12 women who were diagnosed via symptomatic presentation, seven reported that abnormal vaginal bleeding was the trigger symptom, most commonly intermenstrual and postcoital bleeding, and the other five presented with vaginal discharge (n = 3, stages 2a, 2b and 4a), dyspareunia (n = 1, stage 1b1) and abdominal pain (n = 1, stage 1b). Five had had symptoms before the trigger symptom: three had first attributed symptoms (intermenstrual bleeding, dyspareunia, and vaginal discharge), and two had initial symptoms (one had intermenstrual bleeding, the other had postcoital bleeding and dyspareunia).

Table 2 shows the patient durations of symptoms for the 12 women diagnosed via symptomatic presentation. Median patient duration of symptoms from the trigger symptom to first attendance was 1 month (interquartile range (IQR) 0–4 months). Five delayed presenting with their trigger symptom for three or more months. Three of these had abnormal vaginal bleeding (one had intermenstrual bleeding and two had postcoital bleeding), the other two had vaginal discharge and dyspareunia. For four of these women, the trigger symptom was also the first attributed symptom.

**Table 2:** Duration of symptoms for women diagnosed via symptomatic presentation (n = 12)*

**Duration**	**Patient duration of symptoms from trigger symptom**	Patient duration of symptoms that started before the trigger symptom**	
	**n = 12**	**n = 5**	
	**Trigger**	**Initial**	**First attributed**
<1 month	6	1	1
1- < 3 months	1	0	-
3- < 6 months	3	0	-
6- < 12 months	1	0	-
≥12 months	1	1	2
*≥3 months*	*5 (42%)*	*1 (50%)*	*2 (67%)*

* All durations end at first attendance.

** Only includes key symptoms which started prior to the trigger symptom (i.e. initial and first attributed). Women may also have had other symptoms which did not meet key symptom definitions.

Two women had a first attributed symptom earlier than their trigger symptom that started three or more months before their first attendance: one intermenstrual bleeding and one dyspareunia.

For one of the two women who reported initial symptoms, the time from initial symptom to first attendance was two years (stage 4a). Her symptoms were postcoital bleeding and dyspareunia.

Median provider duration of symptoms for the 12 women diagnosed via symptomatic presentation was 3 months (IQR 1–8.5 months, range 0–14 months). For three women, provider duration of symptoms was less than one month, for three women one to three months, and for six women three months or more (for three women it was twelve months or more). All of the women with a provider delay had abnormal vaginal bleeding (4 intermenstrual bleeding, 3 postcoital bleeding, 1 bleeding during pregnancy, 2 heavier/longer periods). Three reported vaginal discharge, two dyspareunia and one abdominal pain.

### Symptoms in women diagnosed via the NHS Cervical Screening Programme

Of the 12 women who were diagnosed via the NHS Cancer Screening Programme, eight (75%) women reported symptoms; five first attributed symptoms (vaginal discharge, postcoital bleeding, intermenstrual bleeding, heavier/longer periods, vaginal discharge, abdominal pain) and three initial symptoms (vaginal discharge, dyspareunia and heavier/longer periods).

### Symptoms in women diagnosed as an incidental finding

All three of the women who were diagnosed as an incidental finding had symptoms, all of which included abnormal vaginal bleeding. Two of the women had first attributed symptoms (bleeding during pregnancy, intermenstrual bleeding and dyspareunia), and one had an initial symptom (postcoital bleeding).

### Inter-rater reliability

There was 100% agreement on the nature (i.e. postcoital bleeding, vaginal discharge etc.) and type (i.e. trigger, first attributed or initial) of key symptoms between the two raters. There was 100% agreement for three of the six dates (i.e. date of diagnosis, date of first attendance, symptom onset dates for trigger, first attributed and initial) examined. For the other three, there was broad agreement although the one rater recorded more precise dates than the other (e.g. rater 1 recorded January 2009, rater 2 recorded October 2008–March 2009). The net result was the same in terms of calculating symptom durations.

### Interview acceptability

Almost all women who expressed a view were positive about their experience of being interviewed and said that they were keen to provide information that would help other young women in the future. Three women reported that they found it upsetting to recount the details of the lead up to their diagnosis or their decision to opt for non-fertility sparing treatment. Most did not find the questions difficult to understand. However, most women said that they found it difficult to remember dates. Two women showed evidence of psychological distress during the interviews that was related to the impact of the cancer on their mental wellbeing rather than the interview itself. We made onward referrals for both for psychological support.

## Discussion

We have developed a methodological approach that allows us to collect, collate and analyse data on the nature and duration of symptoms, and describe the extent of, and risk factors for, patient and provider delay in young women with cervical cancer. We have tested this on 27 women and demonstrated that it permits us to collect meaningful data using reproducible methods. The final interview schedule is designed to elicit a comprehensive account of each woman’s history of presentation from their first symptom that could possibly be due to the cancer, to the time of diagnosis. The interview itself was acceptable to the women and did not appear to cause distress.

A key strength of our study is that we have developed and tested definitions of the pathways to diagnosis, symptom type, durations of symptoms and types of healthcare attendance. We have produced detailed documentation to support our methodological approach, including an interview schedule, interview manual and a data extraction form. Our approach is in keeping with the recommendations made by the Aarhus statement on early diagnosis research [32]. Our definitions allow systematic classification of milestones in the woman’s history of presentation. This was challenging because pathways to cervical cancer diagnosis are often complex, and the gynaecological symptoms occur frequently in young women with non-malignant conditions [19,33,34].

We used several techniques to help promote accurate recall including a timeline, calendar anchoring, and a symptom checklist. Similarly, the interview manual and data extraction schedule promote reproducible data collection as reflected by the high inter-rater reliability agreement.

The main limitation of our approach is that the measure is interview-based and therefore, is time-consuming. Also, our measure of provider delay does not allow us to examine whether delays occurred in primary as opposed to secondary care. This is because our measure collects data from the woman’s point of view and we did not expect women to know details that would help distinguish between primary and secondary care delays. Our definition of the 'trigger’ symptom as the symptom which led to diagnosis, may over- or underestimate patient and provider delays in women who presented with symptoms which did not lead to diagnosis. Although we were unable to present data for some of the risk factors for delay (due to partial data collection), we are confident that we have identified those that are likely to be most relevant to cervical cancer in young women.

Only a small number of women were tested, but our findings provide some early suggestions that there are patient and provider delays in diagnosis of cervical cancer in young women.

We found some suggestion that among the 12 women who presented symptomatically, provider delays were longer than patient delays. For example, one woman who presented to her GP after two weeks of vaginal discharge was not examined but had a positive pregnancy test. At six weeks of pregnancy she presented to accident and emergency with heavy vaginal bleeding, and an ultrasound was performed to check that the pregnancy was viable. At 16 weeks, she presented to her GP with a lump protruding from her vagina and she was referred to physiotherapy for a suspected prolapse. She was diagnosed five months after she first presented, with a stage 2a cancer, following referral from physiotherapy to accident and emergency and on to gynaecology.

Our planned nationwide study of young women diagnosed with symptomatic cancer will use the CCHOPS to examine time to diagnosis in greater detail. This will allow us to confirm the preliminary findings identified during testing.

## Conclusion

We have developed a reliable interview-based measure (Cervical Cancer History of Presentation Schedule) for use in a nationwide study of delayed diagnosis of cervical cancer in young women. This will be crucial to enable us to explore opportunities for earlier intervention, which might improve the diagnosis of cervical cancer at earlier stages. The measure can also be used as a model upon which development of new instruments for systematically measuring delays in diagnosis in other cancers can be based.

## Abbreviations

CCHOPS: Cervical cancer history of presentation schedule; FIGO: International Federation of Gynecology and Obstetrics; GP: General practitioner; IQR: Interquartile range; NHS: National Health Service.

## Competing interests

The authors declare that they have no competing interests.

## Authors’ contributions

All authors participated in the editing of this manuscript and approved the final version for publication. AWWL, AJR and LJLF jointly planned and designed the study and interpreted the data. AWWL recruited the study participants, collected and analysed the study data, performed the interviews, wrote the first draft of the manuscript and contributed to the revising of the manuscript. LJLF extracted data for the inter-rater reliability. AJR and LJLF supervised the interpretation of the analysis, and revised the manuscript. ANR and KSR helped to identify potential study participants and edited and approved the final manuscript.

## Pre-publication history

The pre-publication history for this paper can be accessed here:

http://www.biomedcentral.com/1472-6874/13/45/prepub

## Supplementary Material

Additional file 1: Box 1 Symptom checklist. Click here for file

Additional file 2: Appendix 1 History of presentation of cervical cancer in young women in England. Interview Schedule. Click here for file

Additional file 3: Box 2 Risk factors for patient and provider delays. Click here for file
