# Supplementary material for: Measuring the nature and duration of symptoms of cervical cancer in young women: developing an interview-based approach
Source: BMC Womens Health. 2013 Nov 13;13:45. doi: 10.1186/1472-6874-13-45 (PMC3835395; doi:10.1186/1472-6874-13-45)
Supplement: Additional file 1: Box 1 — Symptom checklist. [file 1472-6874-13-45-S1.doc]

Box 1 Symptom checklist

| - Postcoital bleeding^3 16-21 26^ - Intermenstrual bleeding^3 17 19 21 26^ - Change in periods^16^ - Bleeding during pregnancy - Painful sex - Persistent vaginal discharge^16 17 19 21 26^ - Abdominal or pelvic pain or discomfort^17 19 21^ - Unusual fatigue^16^ |
| --- |
