# Supplementary material for: Measuring the nature and duration of symptoms of cervical cancer in young women: developing an interview-based approach
Source: BMC Womens Health. 2013 Nov 13;13:45. doi: 10.1186/1472-6874-13-45 (PMC3835395; doi:10.1186/1472-6874-13-45)
Supplement: Additional file 2: Appendix 1 — History of presentation of cervical cancer in young women in England. Interview Schedule. [file 1472-6874-13-45-S2.doc]

# History of presentation of cervical cancer in young women i n England

**INTERVIEW SCHEDULE**

# Introduction

*Aim: To make sure the woman understands the study and ensure informed consent.*

- Introduce self and King’s College London research group
- Talk through key points:
- purpose of the study and interview
- length of the interview
- voluntary nature of participation and right to withdraw
- recording of the interview
- access to medical records (GP, hospital and screening)
- Describe confidentiality, and how findings will be reported
- Provide clinical Nurse Specialist contact details
- Answer any questions they have
- Sign consent form

**Ensure the woman understands the purpose of the study and what her participation will involve.**

*Thank you once again for agreeing to take part in the study. I’m the researcher working on this study. I work for King’s College London. We’re interviewing young women in England with cervical cancer. We’re hoping to get a better understanding of the symptoms young women experience and the events that lead them to the doctor and eventually being diagnosed with cervical cancer. If you take part you’ll be contributing to research that could help raise awareness of symptoms of cervical cancer in young women and help them get diagnosed more promptly.*

**Explain the voluntary nature of the study and the right to withdraw.**

*Taking part in the study is up to you and you don’t have to take part. Your medical care will be unaffected by whether or not you take part. You can decide to leave the study at any time. You don’t have to give a reason.*

*What it will involve for you is this interview, which should take around 30 minutes. If you agree, I will write to your GP to let them know you’re taking part and I’ll request a copy of your GP notes, hospital notes and details of any smears you’ve had. Did you get the information sheet that I sent you about the study? Do you have any questions?*

**Answer any questions.**

**Stress confidentiality and explain the purpose of recording.**

*If it’s ok with you, I’d like to record the interview so I don’t have to write it all down while we’re talking and so that I can play it back to make sure I’ve got all the right details. I just want to reassure you that everything we discuss is in the strictest confidence. No one apart from the study team will be able to see any of your information, it won’t be available to the medical team looking after you. You’ve been given a research number so you won’t be identified by name on the recording or on any of our paperwork. We may write some of the things you say in our reports and publications but you personally won’t be identified. I may ask one of my colleagues to listen to the recording to make sure the interviews are being conducted well.*

*We’ll be exploring the events that happened before you were told you had cancer. I imagine that it’s been a difficult time for you, so we’ll go at your pace. If for any reason you’d like to stop or take a break, just let me know. Also, I just want to remind you that if you feel upset after the interview because of the issues raised and would like to talk to someone, I have the contact details of your Clinical Nurse Specialist who is always available for you to speak to, should you want to.*

*If we can just go through the consent form*

**Check if the woman would like you to read out each item or if she is happy to read these quietly herself. Ask her to put her initials in each box if she is happy to consent to the item, and then print her name, the date and sign at the bottom. Permission to access GP notes is optional to study participation, and the woman should tick the appropriate box.**

*Would you like me to read these out to you or would you prefer to read them yourself? Please sign the consent form 2 times, one copy is for you, one for us. Would you like us to tell your GP you’re taking part in the study? And what about your hospital doctor?*

*Was there anything else you wanted to ask before we begin?*

**Reassure the woman that she can take her time to think about the interview questions and that there is no rush.**

*Ok, so as I mentioned, the purpose of this interview is to find out about all of the events that led up to your cancer diagnosis. We’re keen to know when things happened, which can be difficult to remember, but just take as much time as you need and I’ll work with you to try to help you remember.*

## Diagnosis

Aims: To obtain high quality data by starting the interview with the most robust time-points in her history of presentation i.e. her diagnosis. To ease the woman into the interview as these events will be vivid in her memory and easy to recall. To create an anchor point for the recall of events by establishing the time-point at which the woman says she was first told she had cancer.

*Just to start with, can you tell me a bit about what happened when you found out that you had cervical cancer?*

*What did they say was wrong with you?*

*Can you tell me what happened?*

*When did they first tell you that you had cervical cancer?*

*And please correct me if I’m wrong, but your clinical team told me that you’ve had / you’re going to have / you’re having [treatment details] on [date]*

## History of Presentation

Aim: To allow the woman to tell her whole story (history of presentation) and get the ‘big picture’ of what happened in as much detail as possible. To begin filling in the history of presentation timeline.

*Now can we go right back to when you first realised something was wrong...*

*Looking back now, what would you say was the* ***first sign or symptom*** *of your cancer?*

*…and what happened after that? What did you do next?*

*START DRAFTING THE HISTORY OF PRESENTATION TIMELINE*

## Nature, attribution and disclosure of first attributed symptom

Aim: To establish details of the first attributed symptom.

*So with the first symptom xxx,*

- When did you first have this?
- How often did you have it? Was it there all the time or did it come and go?
- How long did you have this for?
- How bad/severe was it?
- What did you think it was due to at the time?
- Did you tell anyone close to you about the symptom? Who was this?

## Actions prompted by the first attributed symptom

Aim: To find out what the first attributed symptom prompted the woman to do (if anything). E.g. self-treat, seek advice from a doctor or nurse, self-examination etc.

*What did you do about the symptom at the time, if anything? And what about later on?*

## Trigger symptom

Aim: To establish the nature of the symptom that first prompted the woman to seek medical advice from a doctor or nurse. .

*What was the symptom(s) that first prompted you to see a doctor or nurse?*

- When did you first have this?
- How often did you have it? Was it there all the time or did it come and go?
- How long did you have this for?
- How bad/severe was it?
- What did you think it was due to at the time?
- Did you tell anyone about this at the time? Who was this?

## Volunteered symptoms

Aim: To find out what other symptoms of cervical cancer the woman experienced that she recalls herself (i.e. unprompted) and attributes to her cancer.

*Did you have any other symptoms after that that you think might be due to the cancer?*

- When did you first have this?
- How often did you have it? Was it there all the time or did it come and go?
- How long did you have this for?
- How bad/severe was it?
- What did you think it was at the time?
- What did you do about the symptom at the time, if anything? And what about later on?
- Did you tell anyone about this at the time? Who was this?

*Are there any other signs or symptoms you noticed before your cancer diagnosis that you think might have been due to your cancer?*

## First attendance

Aim: To establish details of the first attendance. For women who present symptomatically, this will be the first attendance for symptom(s). For women who are diagnosed via NHS Cervical Screening Programmes this will be the consultation for the cervical screening test that led to diagnosis. For women who are diagnosed via another route, this will be the first attendance that led to diagnosis.

***If the woman was diagnosed via the NHS Cancer Screening Programmes:***

*When did you have the smear test that led to your cancer diagnosis?*

*Where did you have the test done?*

***If the woman (1) presented symptomatically or (2) was diagnosed via another route:***

*(1) Thinking about the first time you went to a doctor, nurse or any other healthcare professional for your symptoms…*

*Or*

*(2) Thinking about the consultation that led to your cancer diagnosis...*

### a) Healthcare professional

Aim: To establish what type of healthcare professional the woman saw

*Who did you see?*

### b) Time-point

Aim: To establish the time-point of the consultation

*When was this?*

### c) Consultation details

Aim: To establish where the consultation took place and what happened at the consultation (e.g. details of investigations, examinations, advice or treatment).

- Where was this consultation?
- Can you tell me what happened?
- Did they perform any tests or examinations like a smear, vaginal swabs or pregnancy test?

### d) Action advised

Aim: To find out what advice they were given.

*What advice did they give you?*

- Told was normal
- Referral
- Advice / diagnosis
- Asked to come back for follow up appointment
- Medication (e.g. antibiotics, canesten)
- Change contraceptive pill

*IF THE WOMAN’S FIRST PRESENTATION WAS WITH A NURSE, REPEAT Q7 a)-d) FOR FIRST PRESENTATION WITH A DOCTOR*

*And when did you first see a doctor for your symptom(s)?*

## Other consultations

Aim: To find out details of any other relevant consultations in primary or secondary care.

*Did you ever go to see the doctor (or nurse) again, or any other type of health professional like a nurse or pharmacist, or go to any other clinics like family planning or sexual health for your symptoms?*

***→ If yes, obtain details using Q7 a)-d)***

## First appointment in gynaecology

Aim: To establish the time-point and details of when the woman was seen in secondary care department that is capable of diagnosing cervical cancer.

- Where were you first seen in gynaecology at hospital? Was it general gynaecology, colposcopy or a rapid referral gynaecological oncology clinic?
- When were you first seen in gynaecology or colposcopy?
- What happened at this appointment?

**OK, CAN I JUST QUICKLY CHECK THAT I’VE GOT THIS RIGHT?** *(GO OVER TIMELINE WITH WOMAN AT THE BACK OF THE DATA EXTRACTION SCHEDULE)*

## Prompted symptoms

Aim: To find out if the woman experienced any symptoms of cervical cancer that she hasn’t already mentioned.

*Ok, what I’d like to do now is go through a list of symptoms that we know women can get with cervical cancer. If you could let me know if you experienced any of these, either before or after the symptoms you’ve already told me about* [if symptoms have been reported] *regardless of whether or not you thought they were due to cancer at the time.*

*Looking back, did you have any...*

- Vaginal bleeding between periods
- Vaginal bleeding during or after sex
- Vaginal discharge that lasted more than a couple of days
- Pain or discomfort during sex
- Pain or discomfort in your tummy
- Fatigue that is unusual for you

***For each symptom reported:***

- When did you first experience this?
- How often did you have it? Was it there all the time or did it come and go?
- How long did you have this for?
- How bad/severe was it?
- What did you think it was due to at the time?
- What did you do about it at the time, if anything? And what about later on?

***For initial symptoms:***

- Did you tell anyone about this at the time? Who was this?

*Did you have any other symptoms that we haven’t mentioned?*

## Other health problems

Aim: To find out what other health problems the woman had that may have had an impact on the woman’s experience of, interpretation of, or response to symptoms.

*Now I’d just like to ask you some general questions about your health. We're interested in things that might affect a woman’s decision to go to see a doctor or nurse about symptoms.*

*Have you got any other health problems that restrict your daily activities? For example, stop you doing what you normally do, make you take time off work or if you had to be admitted to hospital.*

- Roughly when did it start / when was this diagnosed?

*Have you had, or do you have, any other gynaecological problems?*

*Have you had any pregnancies, miscarriages or abortions in the last 2 years?*

## Contraception

Aim: To find out if the woman was on any contraception that may have caused or affected symptoms or the woman’s interpretation of symptoms

*Now I’d like to talk about any contraception you may have been using…have you been taking a contraceptive pill, or have a coil, implant or contraceptive injection in the past 2 years?*

***If yes:***

*Which contraception was this?*

*When did you take / have this?*

## Woman’s perception of primary care in relation to her diagnosis of cervical cancer

Aim: To find out what the woman thought of her GP surgery in relation to her experience of being diagnosed with cervical cancer.

*Thinking about the lead up to your cancer diagnosis, how did you find your experience with your GP surgery?*

- Did you feel confident in their ability?
- Did you feel that they listened to your concerns?
- Did you find it easy to get appointments?

## General barriers to seeking GP help

Aim: To find out if the woman perceives any barriers to seeking help from GPs in general (prior to diagnosis).

*Now I’d just like to ask you a question about how you feel about visiting the GP in general. Generally, when you have a health problem, is there anything that would stop you from going to your GP, like say for example…*

- Not wanting to waste the GP’s time
- Finding it difficult to talk to the GP
- Not feeling confident to talk about symptoms with the GP
- Feeling too embarrassed
- Feeling too scared
- Worrying about not getting a female doctor
- Worrying about what the GP might find
- Finding it difficult to get an appointment
- Being too busy to make an appointment
- Having too many other things to worry about
- Anything else?

## Cervical screening history

Aim: To find out whether or not women aged 25 and above ever had a cervical screening test, and if so, the result of their last test.

*Now I’ve like to ask you about your cervical screening history…*

*Have you ever been for a smear test, when you’ve been invited?*

***If no***

*Is there any particular reason why you haven’t had a smear?*

***If yes***

- What was the result of your last test?

## Knowledge and perception of cervical cancer

Aim: To find out women’s level of awareness and knowledge of cervical cancer prior to diagnosis or first experiencing symptoms

*Before you received your cancer diagnosis…*

*Had you heard of cervical cancer?*

***If yes***

- What had you heard about it?
- Did you know what it was?

*Before you had your symptoms [if the woman has reported symptoms], did you know anything about the symptoms of cervical cancer?*

***If yes***

- Which symptoms did you know about?

## Sociodemographic data

Aim: To collect basic sociodemographic data.

*That was the last of the questions on your cancer. Now I’d like to ask you a few bits of general information about you…*

*Can you please look at this list and tell me which of these best represents your ethnic group?*

| **White** |
| --- |
| English / Welsh / Scottish / Northern Irish / British |
| Irish |
| Gypsy or Irish Traveller |
| Any other White background, write below |
|  |
| **Mixed / multiple ethnic groups** |
| White and Black Caribbean |
| White and Black African |
| White and Asian |
| Any other Mixed/multiple ethnic background, write below |
|  |
| **Asian / Asian British** |
| Indian |
| Pakistani |
| Bangladeshi |
| Chinese |
| Any other Asian background, write below |
|  |
| **Black / African / Caribbean / Black British** |
| African |
| Caribbean |
| Any other Black / African / Caribbean background, write below |
|  |
| **Other ethnic group** |
| Arab |
| Any other ethnic group, write below |
|  |

*At what age did you leave full time education?*

*Do you have any children?*

*What were your living arrangements before you received your cancer diagnosis?*

*Were you single or in a relationship before you received your cancer diagnosis?*

*What is your GP’s name and address?*

*OK, that is all I need to ask you. Do you want to add anything or ask me any other questions about the research?*

*Many thanks once again for agreeing to be interviewed.*

**~~ END OF INTERVIEW ~~**
