# Supplementary material for: Measuring the nature and duration of symptoms of cervical cancer in young women: developing an interview-based approach
Source: BMC Womens Health. 2013 Nov 13;13:45. doi: 10.1186/1472-6874-13-45 (PMC3835395; doi:10.1186/1472-6874-13-45)
Supplement: Additional file 3: Box 2 — Risk factors for patient and provider delays. [file 1472-6874-13-45-S3.doc]

Box 2 Risk factors for patient and provider delays

| **Key symptoms**  Nature of symptom  Severe or not  Perceived by the woman as serious or not  Disclosure of symptom to others  **Demographic factors**  Ethnic group  Age <25 at diagnosis (i.e. not offered routine cervical screening)  Index of multiple deprivation of lower superoutput area of residence calculated from postcode  Living arrangements (i.e. who they live with)  Relationship status (i.e. single or in a relationship)  Has children  Age left full-time education  **Other gynaecological factors**  Previous gynaecological problems  Use of hormonal contraception or intrauterine contraceptive devices  Pregnancy  Whether previously attended for cervical screening on the NHS Cervical Screening Programme  Result of last cervical cytology (if aged 25+)  **Knowledge and beliefs**  Perceived barriers to attending her GP  Prior knowledge of cervical cancer  **Medical history (non-gynaecological)**  Chronic conditions that restrict daily activities |
| --- |
